# Supplementary material for: Effect of Polydeoxyribonucleotide (PDRN) Treatment on Corneal Wound Healing in Zebrafish (Danio rerio)
Source: Int J Mol Sci. 2022 Nov 4;23(21):13525. doi: 10.3390/ijms232113525 (PMC9659220; doi:10.3390/ijms232113525)
Supplement: Supplementary file 1 [file ijms-23-13525-s001.zip › ijms-1940078-supplementary.pdf]

International Journal of
Molecular Sciences

MDPI

Supplementary Materials:

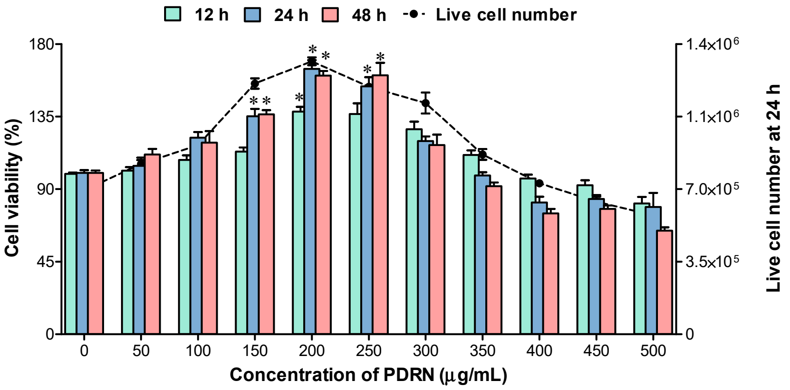

Figure S1: Effect of PDRN on cell viability and live cell number in HDFs. The results are presented as percent of cell viability in control (0 μg/mL of PDRN) in each time point. The viability of untreated control cells was taken as 100%. Results are presented as means ± SD (n = 3). *P < 0.05 compared to the control (0 mg/mL of PDRN) as tested by Student's t-test.

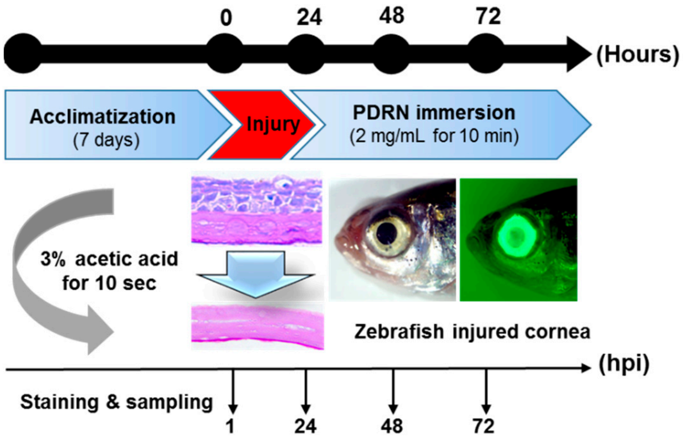

Figure S2: Overview of the experimental design of acetic acid induced corneal injury and PDRN treatment in zebrafish.

Table S1: Gene specific primers used in this study.

Copyright: © 2022 by the authors. Licensee MDPI, Basel, Switzerland. This article is an open access article distributed under the terms and conditions of the Creative Commons Attribution (CC BY) license (https://creativecommons.org/licenses/by/4.0/).

Gene	Accession number	Primer name	Forward primer
Adenosine A2a receptor a (*adora2a.1*)	AY945800.1	adora2a.1-F	GCTCCACCCTACAGAAAGAAG
		adora2a.1-R	CAGGGCAGAACAGAGTGAAA
Adenosine A2a receptor b (*adora2ab*)	NM_001040036.1	adora2ab-F	GAGCTCCGTCTTCAGTTTGT
		adora2ab-R	GCAATGATTCCTTTGGCTCTTC
Adenosine A1 receptor b (*adora1b*)	NM_001128584.1	adora1b-F	AGAAGTTTCGGACTGCCTTT
		adora1b-R	AGCGAAGTAGTGGTTGTTATCC
Adenosine A2b receptor (*adora2b*)	NM_001039813.2	adora2b-F	CCATCAACTCTACGCTCAAGAA
		adora2b-R	GACCACAGACCGATGCTTATAG
Tumor necrosis factor- α (*tnf-α*)	AY427649	tnf-α-F	AGAAGGAGAGTTGCCTTTACCGCT
		tnf-α-R	AACACCCTCCATACACCCGACTTT
Matrix metalloproteinase 9 (*mmp9*)	AY151254	mmp9-F	TTTGCCCTGATCGTGGATAC
		mmp9-R	GGGAAACCCTCCACGTATTT
Matrix metalloproteinase 13 (*mmp13*)	AF506756	mmp13-F	GAGAAGGTTTGGGCTCTCTATG
		mmp13-R	TGAGTTGCTGTCTTCCTTGTAG
Transforming growth factor, β1 (*tgfβ1*)	XM_687246	tgfβ1-F	CCCAAGGAACCAGAAGTAGAAG
		tgfβ1-R	GGATCTTCTATGGTGTGCTGAA
Paired box 6a (*pax6a*)	NM_131304.1	pax6a-F	CCTCAAGTCACATACCCATCAG
		pax6a-R	GAGCCTGACGTAAAGGATACTG
Paired box 6b (*pax6b*)	NM_131641.1	pax6b-F	AAGTGGAGGAGAGAGGAGAAA
		pax6b-R	TGATAGACGCTGGTGTTGAAG
Heat shock protein 70 (*hsp70*)	AB062116.1	hsp70-F	CATGGTCCTGGTGAAGATGAA
		hsp70-R	GTCTGTGGGACTCGTTGAAATA
Heat shock protein 90 (*hsp90ab.1*)	NM_131310.3	hsp90ab.1-F	GAAGAGGAGAAGGCAGAGAAAG
		hsp90ab.1-R	CGAGCCGACATCTTCAATCT
Kruppel-like factor 4 (*klf4*)	NM_001113483	klf4-F	CCTCCACGGGAAATTTGTAGTC
		klf4-R	CCACTATACAGCCGCAGTTC
Mucin 2.1 (*muc2.1*)	NC_007136.7	muc2.1-F	AATATGCCTTGCGGAACAAC
		muc2.1-R	GTGCTGAGGTTGCAGAATGA
Mucin 5.1 (*muc5.1*)	XM_009297795.1	muc5.1-F	TGGCAACTTGGCTGATGATA
		muc5.1-R	TCGTCACACGGACCAGTAGA
Mucin 5.2 (*mus5.2*)	XM_009297793.1	mus5.2-F	GGTGTCTGTTCCGATCAATC
		mus5.2-R	TCATCCTTGTCGCCATTGTA

β-actin	AF025305	β-actin- F	AATCTTGCGGTATCCACGAGACCA
		β-actin- F	TCTCCTTCTGCATCCTGTCAGCAA
